# Somatic mutation profiling and *HER2* status in *KRAS*-positive Chinese colorectal cancer patients

**DOI:** 10.1038/s41598-019-53039-y

**Published:** 2019-11-15

**Authors:** Zhouhuan Dong, Linghong Kong, Zhiyi Wan, Fengwei Zhu, Mei Zhong, Yali Lv, Po Zhao, Huaiyin Shi

**Affiliations:** 1 Chinese PLA General Hospital, Department of Pathology, Beijing, 100853 China; 2 ChuiYangLiu Hospital affiliated to Tsinghua University, Department of Pathology, Beijing, 100022 China

**Keywords:** Predictive markers, Tumour biomarkers

## Abstract

*KRAS* is an independent negative predictor for anti-epidermal growth factor receptor (anti-EGFR) treatment in colorectal cancers (CRCs). However, 30% to 50% of CRC patients are *KRAS*-positive and do not benefit from anti-EGFR therapy. In this study, we investigated the mutational features and clinical significance of *KRAS*-positive Chinese CRC patients. A total of 139 Chinese CRC patients who received clinical *KRAS* testing (Sanger sequencing) were examined by immunohistochemistry (IHC) and fluorescence *in situ* hybridization (FISH). Fifty *KRAS*-positive specimens were further detected by next-generation sequencing (NGS). The most prevalent mutation in *KRAS* was G12D (46%), followed by G12V (20%), and G13D (18%). In addition to *KRAS*, 72 unique alterations in another 12 genes were also detected. The most common mutated genes were *TP53* (62%), *APC* (46%), and *PIK3CA* (22%). The proportion of *HER2* amplifications in *KRAS*-positive CRC patients was 4.4%, which was lower than that in *KRAS* -negative CRC patients (14.3%). No relationship was found between *HER2* amplification and *KRAS* status (p = 0.052). However, the odds ratio is very low (0.279). In addition, these gene mutations were not significantly associated with age, sex, tumor size, lymph node metastasis, mismatch repair–deficient, or tumor differentiation. However, *TP53* mutations were more prevalent in colon cancer with *KRAS* mutations than in rectal cancer (75.0% vs 28.6%, respectively, p = 0.004). The negative predictive value of the IHC analysis for predicting *HER2* amplification reached to 98.39%, while the positive predictive value reached only 50%. Overall, the mutation profiling of Chinese CRC patients with *KRAS* mutations is different from that of Western CRC patients. Our results will help us to understand the molecular features of Chinese CRC patients.

## Introduction

In 2018, colorectal cancer (CRC) was the third most common malignancy worldwide^1^. In China, CRC was the fourth most commonly diagnosed cancer and the fifth leading cause of cancer-related death in 2015^2^. In addition, the incidence of CRC steadily increased from 2000 to 2013^3^.

The pathogenesis of CRC is influenced by the local colonic environment and the individual’s genetic background. A large number of cancer-relevant genes with specific clinical significance have been identified, such as epidermal growth factor receptor *(EGFR*), *KRAS, ERBB2, BRAF*, and *PIK3CA*^4^. The EGFR pathway, which is activated by mutations in *KRAS, NRAS, BRAF*, and *PIK3CA*, plays a crucial role in the regulation of cell proliferation, apoptosis, and angiogenesis in CRC^4^. Therefore, mutations in *KRAS, NRAS, BRAF*, and *PIK3CA* are important predictive and prognostic markers for anti-EGFR therapy^5–7^. Current guidelines have recommended that the mutation status of *KRAS, NRAS*, and *BRAF* should be tested when considering anti-EGFR treatment^8–10^. However, a rapidly growing list of genes should be examined for improving CRC management, such as human epidermal growth factor receptor 2 (*ERBB2*) and *ERBB3*.

*ERBB2*, also known as *HER2*, encodes a transmembrane receptor tyrosine kinase^11^. It is a target for patients with breast cancer or gastric cancer^12,13^. In CRC, HER2 overexpression and amplification have also been used as potential therapeutic targets^14–16^. In addition, HER2 overexpression will cause resistance to anti-EGFR therapy^17^. Although a few studies have reported the incidence rate of HER2 overexpression or amplification in CRC, it varies considerably, ranging from 0% to 83%^18–22^. Moreover, *HER2* status in Chinese CRC has not yet been fully studied.

The relationship between *KRAS* and *HER2* status also remains to be elucidated. One report showed that *KRAS* mutations and *HER2* amplification were mutually exclusive^22^, while another study showed no relationship between *HER2* amplification and *KRAS* mutations^18^. Thus, anti-HER2 therapy, like trastuzumab, may be a possible treatment option for CRCs with *KRAS* mutations^18^.

HER2 overexpression is usually detected by the immunohistochemistry (IHC) analysis of HER2 protein or the fluorescence *in situ* hybridization (FISH) analysis of gene amplification. Although several IHC scoring systems of HER2 for CRC have been provided^23,24^, there is currently no broad consensus on the diagnostic criteria. Moreover, the concordance between the results of IHC and FISH has yet to be verified.

Therefore, we investigated the mutational features and clinical significance of *KRAS*-positive Chinese CRCs by next-generation sequencing (NGS), IHC, and FISH. We further explored the relationship between *KRAS* and *HER2* and the concordance between the results of IHC and FISH for *HER2* testing in CRCs.

## Results

### Mutational spectrum of Chinese CRC patients with KRAS mutations

**Figure 1:**
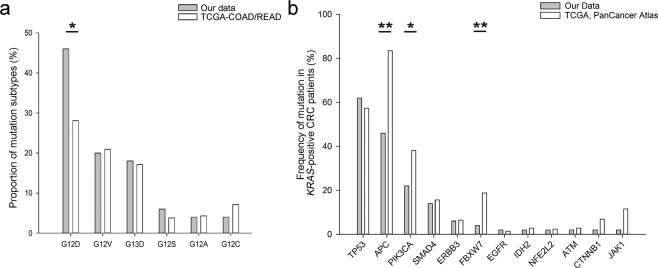
Mutational landscape of 50 *KRAS*-positive Chinese colorectal cancer (CRC) patients and Western patients. (**a**) Proportions of *KRAS* mutation subtypes. The data of Western patients were obtained from TCGA-COAD and TCGA- READ (n = 209). G12D, OR = 2.208, 95% confidence intervals (CI) = 1.157–4.214, p = 0.025. (**b**) Distribution of somatic mutated genes other than *KRAS*. The data of Western patients with *KRAS* mutations were obtained from the TCGA PanCancer Atlas (n = 218). *APC*, OR = 0.168, 95% CI = 0.087–0.326, p = 0.000; *PIK3CA*, OR = 0.459, 95% CI = 0.223–0.945, p = 0.033; *FBXM7*, OR = 0.180, 95% CI = 0.042–0.77, p = 0.009. Statistically significant differences were analyzed by Fisher’s exact test; *P < 0.05, **P < 0.01.

Based on the results of *KRAS* by Sanger sequencing, fifty *KRAS*-positive specimens were further detected by NGS using SGI OncoAim™DNA panel (Singlera Genomics, Shanghai, China), an amplification-based enrichment method that covers more than 6000 known hotspots, yielding a median depth of 816× and a median uniformity of 95.25%. According to the quality control standards, all these specimens produced qualified sequencing data. All 50 samples harbored a mutation in exon 2 of the *KRAS* gene, in accordance with the results of Sanger sequencing. The most prevalent mutation in *KRAS* was G12D (46%), followed by G12V (20%) and G13D (18%) (Fig. 1a).

In addition to *KRAS* mutation, at least one other alteration was detected in 48 patients (96%). A total of 72 unique alterations in 12 other genes were identified, averaging 1.44 alterations per sample (range, 0–5). Most patients harbored 1–3 mutations, while only one patient harbored 5 concomitant mutations. In addition to *KRAS*, the mutated genes with a frequency ≥5% were *TP53* (62%), *APC* (46%), *PIK3CA* (22%), *SMAD4* (14%), and *ERBB3* (6%) (Fig. 1b). The distribution of all gene mutations in all patients is shown in Table S2. In addition, no mutation (SNVs or InDels) was detected in *NRAS, HRAS, BRAF*, or *ERBB2*. Some hotspot mutations were detected, such as R1450* and T1556fs*3 in *APC*, and R361C/H in *SMAD4*. These mutations were all observed in 10% (5/50) patients. The *HER2* status of these 50 *KRAS*-positive specimens was assessed using IHC and FISH. However, *HER2* amplification was not detected.

Finally, we compared somatic mutation profiling in Chinese CRCs with *KRAS* mutations to that in Western CRCs with *KRAS* mutations. For analysis of *KRAS* mutation subtypes, the data on Western CRC patients were obtained from the TCGA-COAD and TCGA- READ (https://portal.gdc.cancer.gov/exploration). There are currently 209 CRCs with *KRAS* mutations, which include 176 colon adenocarcinomas and 33 rectal adenocarcinomas. The specific types of *KRAS* mutations in Chinese patients were slightly different from those in Western patients (Fig. 1a). The proportion of G12D in Chinese CRC patients with *KRAS* mutations was significantly higher than that in Western CRC patients (Fig. 1a, Fisher’s exact test, OR = 2.208, 95% CI = 1.157–4.214, p = 0.025). For comparative analysis of somatic mutation spectrum, according to the TCGA PanCancer Atlas (http://www.cbioportal.org/study/summary?id = coadread_tcga_pan_can_atlas_2018), the molecular spectrum of 218 CRC patients with *KRAS* mutations was available. The mutation profiling of Chinese CRC patients with *KRAS* mutations was also different from that of Western CRC patients (Fig. 1b). The mutation frequencies of *APC, PIK3CA, and FBXW7* in Chinese CRC patients with *KRAS* mutations were significantly lower than that in Western CRC patients with *KRAS* mutations (Fig. 1b, Fisher’s exact test).

### Correlation of gene mutations with clinicopathological features

**Figure 2:**
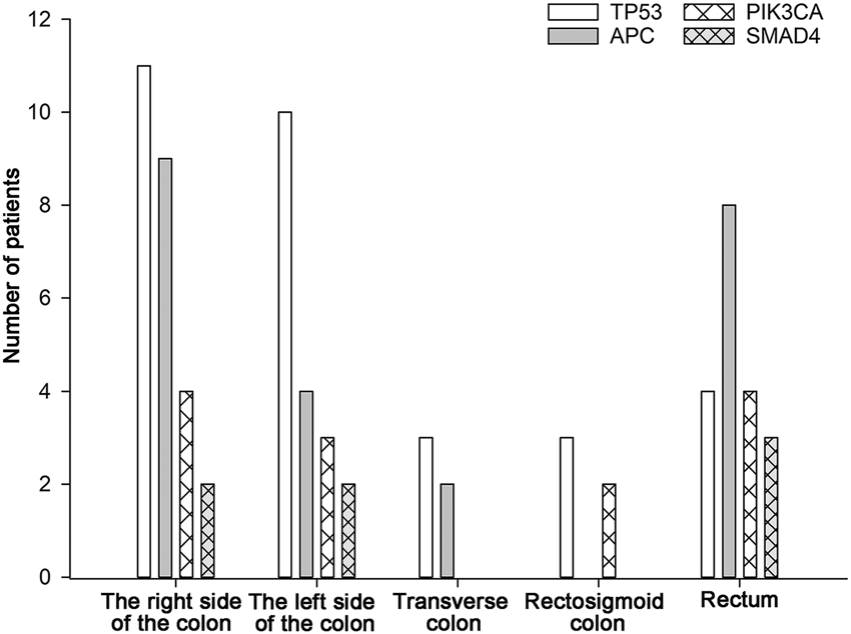
Tumor site distribution of *TP53, APC, PIK3CA*, and *SMAD4*.

In these samples, the prevalence of CRC was higher in males (n = 30) than in females (n = 20). The locations of the primary tumors included the left side of the colon (n = 16), the right side of the colon (n = 14), the transverse colon (n = 3), the rectosigmoid colon (n = 3), and the rectum (n = 14). In addition to *KRAS* mutation, a summary of the relationships among the mutated genes with a frequency ≥10% and various clinicopathological features is shown in Table 1 and Fig. 2. No significant relationship was observed between these four mutated genes and age, gender, tumor size, tumor differentiation, mismatch repair–deficient (dMMR), or lymph node metastasis. However, *TP53* mutations were significantly more prevalent in tumors in the colon than in tumors in the rectum (27/36, 75% vs 4/14, 28.57%, respectively; p = 0.004, Table 1).

**Table 1:** Correlation between the mutated genes with frequency ≥10% and clinicopathological parameters in *KRAS*-positive CRC patients.

Clinicopathological features	number	TP53	p value	APC	p value	PIK3CA	p value	SMAD4	p value
Gender									
Male	30	19	1.000	13	0.774	8	0.489	5	0.687
Female	20	12		10		3		2	
Age (years)									
≥50	46	30	0.147	20	0.322	10	1.000	7	1.000
<50	4	1		3		1		0	
Tumor site									
Colon	36	27	0.004	15	0.361	8	1.000	4	0.384
Rectum	14	4		8		3		3	
Differentiation									
Well/Moderate	12	7	1.000	3	0.186	3	1.000	2	1.000
Poor	38	23		20		8		5	
Lymph node metastasis									
Positive	33	21	0.767	15	1.000	8	0.728	4	0.677
Negative	17	10		8		3		3	
Tumor size									
≤3 cm	11	7	0.551	7	0.304	4	0.403	2	0.200
3~5 cm	25	17		9		4		5	
>5 cm	14	7		7		3		0	
KRAS mutation									
G12	41	28	0.067	19	1.000	11	0.177	7	0.325
G13	9	3		4		0		0	
dMMR/pMMR									
pMMR	44	29	0.184	19	0.395	10	1.000	7	0.576
dMMR	6	2		4		1		0	

Abbreviations: dMMR = mismatch-repair deficiency; pMMR = mismatch repair–proficient.

### Relationship between *HER2* and *KRAS* status

To further assess the consistency of IHC and FISH for *HER2*, another 89 unselected colorectal cancer patients were enrolled and analyzed. The results are shown in Table S3. IHC 0/1+ was found in 62 of 139 patients (44.60%), IHC 2+ was found in 69 patients (49.64%), and IHC 3+ was found in 8 patients (5.76%). Representative images of IHC and FISH analyses are shown in Fig. S1. In comparison with the FISH results, the positive and negative predictive values of the IHC analysis for predicting *HER2* amplification were 50.00% and 98.39%, respectively. In addition, of 69 samples with equivocal IHC results, only 6 (8.70%) were confirmed as harboring *HER2* amplification by FISH.

To assess the relationship between *HER2* and *KRAS* status, a Fisher’s exact test was performed on these 139 CRCs. No significant relationship was found between *HER2* amplification and *KRAS* status (OR = 0.279, 95% CI = 0.077–1.006, p = 0.052, Table 2).

**Table 2:** Comparison of *HER2* and *KRAS* status in 139 primary CRC patients.

	KRAS+	KRAS−	Odds Ratio (95% CI)	P
HER2 FISH+	4	7	0.279 (0.077–1.006)	0.052
HER2 FISH−	86	42		

Abbreviations: CI = confidence interval.

## Discussion

After the comprehensive molecular characterization of colorectal cancer was reported by the TCGA^22^, an increasing amount of data have accumulated rapidly in different genetic or clinical backgrounds^25–28^. Molecular testing has become increasingly significant for the treatment of CRCs. Mutations in genes such as *KRAS, NRAS*, and *BRAF* have become important negative predictive markers for EGFR-targeted therapies^5,6,9^. However, 30% to 50% of CRC patients were *KRAS* positive^22,25–27^, which suggests that a considerable number of patients do not benefit from anti-EGFR therapy^29^. Therefore, it is particularly necessary to understand the genetic profiling of patients with *KRAS* mutations for their prediction and prognosis. In this study, 50 *KRAS*-positive Chinese CRCs and 89 additional unselected Chinese CRCs were examined by NGS, IHC, and FISH. The correlations of these genetic mutations with clinicopathological features were also assessed.

All 50 *KRAS*-positive samples (Sanger sequencing) were also positively detected by NGS, showing that the NGS method used in this study is highly accurate. No mutation was detected in *NRAS, HRAS*, or *BRAF*, consistent with the report that the genes in the *RAS* family are mutually exclusive^22^. In addition to *KRAS*, *TP53* was the most frequently mutated gene (62%), consistent with published data^22,26^, followed by *APC* (46%), *PIK3CA* (22%), *SMAD4* (14%), and *ERBB3* (6%). In addition, there was a higher co-mutation rate of *KRAS* and *TP53* in colon cancer than in rectal cancer.

*APC*, as a gatekeeper gene in CRC, is mutated in 50%-80% of unselected CRC patients in Western countries^22,30,31^. Based on the data from the TCGA PanCancer Atlas, the frequency of *APC* mutations was 83.5% in *KRAS*-positive CRC patients, which was significantly higher than that in our study. However, considering the influence of factors such as the small sample size and the different sequencing technology, this difference needs further verification. For example, *APC* mutations are more difficult to detect in a hotspot panel for this cohort compared to the entire exonic sequence for the TCGA.

During the last decade, HER2 has been investigated as a therapeutic target in metastatic colorectal cancer (mCRC) in several small studies^15,16^. Although the results of MyPathway indicated that patients with *HER2*-amplified, *KRAS*-mutant tumors were not sensitive to anti-HER2 therapy, this finding is more likely due to the lower *HER2* copy numbers in these patients^32^. The incidence of *HER2* amplification and/or protein overexpression ranges from 1% to 6% in the unselected population^32–34^, which is lower than that in patients with breast cancer (~25%) or gastric cancer (13–22%)^35,36^. In our study, *HER2* amplification was observed in 4.4% of CRC patients with *KRAS* mutations (n = 90), which is in accordance with previous reports^18,32^. Although the incidence of *HER2* amplification in *KRAS* negative CRCs reached up to 14.3%, there was no statistical significance between *HER2* amplification and *KRAS* status (p = 0.052). This result is also consistent with the earlier report^18^. However, the OR is very low (0.279), and the p-value is right at the cut-off point. There is also a previous report for *KRAS* being mutually exclusive with *HER2* amplification^37^. Therefore, we speculated that there is reduced likelihood of having a *HER2* amplification event in CRCs with *KRAS* mutations.

HER2 overexpression is usually detected by the IHC analysis of the HER2 protein or the FISH analysis of gene amplification. The results of these two methods are usually consistent in breast and gastric cancers^11,14^. However, the consistency of these two methods in CRCs is unknown, which is partly because that the criteria for HER2-positivity in CRCs has not yet reached a broad consensus, although some pathologists believe that the criteria for HER2-positivity in CRC should differ from that in breast or gastric cancer^14^. In this study, as in the previous report^32^, the scoring was performed according to the guidelines of HER2 testing in gastric cancer^24^. In comparison with the FISH results, the negative predictive value of the IHC analysis for predicting *HER2* amplification reached 98.39%, while the positive predictive value reached only 50%. In addition, for 69 samples with an IHC score of 2, only 6 (8.70%) harbored *HER2* amplification confirmed by FISH. These two ratios were significantly lower than those in breast or gastric cancer. This difference may be explained by the criteria used for HER2-positivity in gastric cancer, which is not particularly suitable for the assessment of HER2 scoring in CRC. More data are needed to correct the IHC testing guidelines for HER2 in CRC.

In the study reported by Park *et al*.^38^, only 7.4% (2/27) CRC with HER2 IHC scores of 3+ were *HER2* amplification. However, in the report described by Wang *et al*.^39^, 11.8% (12/102) of CRCs with HER2 IHC scores of 2+ and in 82.8% (24/29) of CRCs with HER2 IHC scores of 3+ were identified as *HER2* amplification. One explanation for these differences may be that antibodies used for IHC or positive criteria for FISH varied among research groups. In the study reported by Park *et al*.^38^, the IHC staining was performed using a polyclonal antibody of uncertain reliability, which may produce more HER2 IHC scores of 3+. In the study reported by Wang *et al*.^39^, the positive criteria for *HER2* amplification was defined as a HER2/CEP17 ratio of ≥2.0. While, *HER2* amplification was defined as a HER2/CEP17 ratio of ≥2.2 in our study.

In conclusion, we described a mutational profile of Chinese CRCs with *KRAS* mutations by multiple genes and performed an exploratory analysis to make clinical correlations. The mutation profiling of Chinese CRC patients with *KRAS* mutations is maybe different from that of Western CRC patients. We also assessed the consistency of IHC and FISH analyses for HER2 in CRCs. These findings will help us understand the molecular subtypes of Chinese CRCs and refine management decisions for individual patients. However, the reference values of mutation frequencies of different genes were limited due to the small number of samples and mutation-detecting method. More CRC samples are needed for comprehensive genetic testing.

## Methods

### Clinical patients and specimens

We retrospectively investigated 139 CRC patients who received clinical *KRAS* testing (Sanger sequencing) at the Chinese PLA General Hospital (Beijing, China) between May 2015 and October 2017. Sections from formalin-fixed paraffin-embedded (FFPE) tissue samples were stained with hematoxylin–eosin and examined by experienced pathologists to ensure a tumor content ≥20%. All 139 specimens were detected by IHC and FISH, while 50 *KRAS*-positive specimens were further detected by NGS. This study was conducted with the approval of the Ethics Committee of the Chinese PLA General Hospital, and informed consent was obtained from all patients. The methods were carried out in accordance with approved guidelines.

### DNA extraction

DNA was extracted using a QIAamp DNA FFPE Tissue Kit (Qiagen, Hilden, Germany) according to the manufacturer’s protocol. The quantity and quality of the isolated DNA were tested using a Qubit 3.0 fluorimeter (Life Technologies, Eugene, Oregon, USA).

### Sanger sequencing

Primers were designed to amplify exon 2 of the *KRAS* gene to investigate the mutational status of codons 12 and 13. The forward and reverse oligonucleotide primers were: 5′- ATTACGATACACGTCTGCAGTCAACTG-3′ and 5′-CAATTTAAACCCACCTATA ATGGT-3′, respectively. Purified PCR products were sequenced on an ABI 3730xL sequencer using a BigDye Terminator v3.1 Sequencing Kit (Applied Biosystems, Waltham, MA, USA) according to the manufacturer’s protocol.

### NGS analysis

DNA libraries and sequencing were performed using an SGI OncoAim™DNA Kit (Singlera Genomics, Shanghai, China) according to the manufacturer’s protocol^25^. The SGI OncoAim™ DNA Panel covers more than 6000 hotspots (single nucleotide variants (SNVs) and short insertions and deletions (InDels)) in 59 genes (Table S1). Next, 150 bp paired-end sequencing was performed on the Illumina MiSeq (Illumina, Hayward, CA, USA). Bioinformatics analysis of NGS sequencing data was performed according to the pipeline of the SGI OncoAim™ DNA Kit. Sequencing data with a minimum median read depth of 500× and a minimum uniformity of 80% were considered qualified. Mutations with a mutation allele frequency (MAF) ≥5% were reported.

### IHC analysis

Sections of FFPE tissue (4 um thick) were obtained. HER2 immunostaining was performed using a PATHWAY anti-HER2/neu (4B5; rabbit monoclonal; predilution; Ventana Medical Systems, Tucson, AZ, USA) antibody and an ultraView Universal DAB Kit (Ventana Medical Systems) on an automatic immunostainer (BenchMark XT, Ventana Medical Systems), according to the manufacturer’s instructions. HER2 immunoreactivity was evaluated by two pathologists according to the scoring system described by Josef Ru¨ schoff *et al*.^24^ as follows: 0, no reactivity or membrane staining in <10% of tumor cells; 1+, faint/barely perceptible membranous reactivity in ≥10% of tumor cells; 2+, weak-to-moderate complete, basolateral, or lateral membranous reactivity in ≥10% of tumor cells; and 3+, strong basolateral, or lateral membranous reactivity in ≥10% of tumor cells.

### FISH analysis

FISH analysis was performed using the PathVysion *HER2* DNA Probe Kit (Abbott Molecular Inc, Des Plaines, IL, USA), according to the manufacturer’s protocols. FISH signal assessment was performed by visual counting using an epifluorescence microscope (BX53F; Olympus, Tokyo, Japan). At least 50 tumor cells per case with a minimum of one signal for the *HER2* gene and centromere 17 were randomly selected, and the mean *HER2* and centromere 17 count was calculated. Amplification was defined as a *HER2/CEP17* ratio of ≥2.2 in 20 tumor nuclei. The equivocal cases (ratio: 1.8 to 2.2) were recounted in at least 20 nonoverlapping nuclei of different tumor cells at a second target area, and a new *HER2/CEP17* ratio was recalculated.

### Statistical analysis

Statistical analysis was carried out with SPSS 19.0 statistical software (SPSS, Inc., Chicago, IL, USA). The Fisher’s exact test was used to compare the rates among groups with different features. Odds ratios (OR) and their 95% confidence intervals (CI) were calculated. Statistical tests were two-sided, and p < 0.05 was considered significant.

## Supplementary information

Somatic mutation profiling and HER2 status in KRAS-positive Chinese colorectal cancer patients

## Data Availability

All data generated or analyzed during this study are included in this published article and its supplementary information files.

## References

[1] Bray F (2018). Global cancer statistics 2018: GLOBOCAN estimates of incidence and mortality worldwide for 36 cancers in 185 countries. Ca: A Cancer Journal for Clinicians.

[2] Chen W (2016). Cancer statistics in China, 2015. Ca: A Cancer Journal for Clinicians.

[3] Chen W (2017). Cancer incidence and mortality in China, 2013. Cancer Letters.

[4] Berg M, Soreide K (2012). EGFR and downstream genetic alterations in KRAS/BRAF and PI3K/AKT pathways in colorectal cancer: implications for targeted therapy. Discovery Medicine.

[5] Gattenlohner S, Germer C, Muller-Hermelink HK (2009). K-ras mutations and cetuximab in colorectal cancer. The New England Journal of Medicine.

[6] De Roock W (2010). Effects of KRAS, BRAF, NRAS, and PIK3CA mutations on the efficacy of cetuximab plus chemotherapy in chemotherapy-refractory metastatic colorectal cancer: a retrospective consortium analysis. The Lancet. Oncology.

[7] Sartore-Bianchi A (2009). PIK3CA mutations in colorectal cancer are associated with clinical resistance to EGFR-targeted monoclonal antibodies. Cancer Research.

[8] Van Cutsem E (2016). ESMO consensus guidelines for the management of patients with metastatic colorectal cancer. Annals of Oncology.

[9] Benson AB (2017). Colon Cancer, Version 1.2017, NCCN Clinical Practice Guidelines in Oncology. Journal of the National Comprehensive Cancer Network.

[10] Benson AB (2018). Rectal Cancer, Version 2.2018, NCCN Clinical Practice Guidelines in Oncology. Journal of the National Comprehensive Cancer Network.

[11] Pyo JS, Sohn JH, Kim WH (2016). Concordance rate between HER2 immunohistochemistry and *in situ* hybridization in gastric carcinoma: systematic review and meta-analysis. The International Journal of Biological Markers.

[12] Paik S (1990). Pathologic findings from the National Surgical Adjuvant Breast and Bowel Project: prognostic significance of erbB-2 protein overexpression in primary breast cancer. Journal of Clinical Oncology.

[13] Bang YJ (2010). Trastuzumab in combination with chemotherapy versus chemotherapy alone for treatment of HER2-positive advanced gastric or gastro-oesophageal junction cancer (ToGA): a phase 3, open-label, randomised controlled trial. Lancet.

[14] Greally M, Kelly CM, Cercek A (2018). HER2: An emerging target in colorectal cancer. Current Problems in Cancer.

[15] Sartore-Bianchi A (2016). Dual-targeted therapy with trastuzumab and lapatinib in treatment-refractory, KRAS codon 12/13 wild-type, HER2-positive metastatic colorectal cancer (HERACLES): a proof-of-concept, multicentre, open-label, phase 2 trial. The Lancet Oncology.

[16] Richman SD (2016). HER2 overexpression and amplification as a potential therapeutic target in colorectal cancer: analysis of 3256 patients enrolled in the QUASAR, FOCUS and PICCOLO colorectal cancer trials. The Journal of Pathology.

[17] Yonesaka K (2011). Activation of ERBB2 signaling causes resistance to the EGFR-directed therapeutic antibody cetuximab. Science Translational Medicine.

[18] Lee W-S (2014). Comparison of HER2 expression between primary colorectal cancer and their corresponding metastases. Cancer Medicine.

[19] Schuell B, Gruenberger T, Scheithauer W, Zielinski C, Wrba F (2006). HER 2/neu protein expression in colorectal cancer. BMC Cancer.

[20] Ross JS, McKenna BJ (2001). The HER-2/neu oncogene in tumors of the gastrointestinal tract. Cancer Investigation.

[21] Conradi LC (2013). Frequency of HER-2 positivity in rectal cancer and prognosis. The American Journal of Surgical Pathology.

[22] The Cancer Genome Atlas N (2012). Comprehensive molecular characterization of human colon and rectal cancer. Nature.

[23] Valtorta E (2015). Assessment of a HER2 scoring system for colorectal cancer: results from a validation study. Modern Pathology.

[24] Ruschoff J (2012). HER2 testing in gastric cancer: a practical approach. Modern Pathology.

[25] Wang Y (2018). Performance validation of an amplicon-based targeted next-generation sequencing assay and mutation profiling of 648 Chinese colorectal cancer patients. Virchows Archiv.

[26] Chang YS, Chang SJ, Yeh KT, Lin TH, Chang JG (2013). RAS, BRAF, and TP53 gene mutations in Taiwanese colorectal cancer patients. Onkologie.

[27] Zhang J (2015). Molecular spectrum of KRAS, NRAS, BRAF and PIK3CA mutations in Chinese colorectal cancer patients: analysis of 1,110 cases. Scientific Reports.

[28] Chiu JW (2018). Molecular Profiling of Patients With Advanced Colorectal Cancer: Princess Margaret Cancer Centre Experience. Clinical Colorectal Cancer.

[29] Linardou H (2008). Assessment of somatic k-RAS mutations as a mechanism associated with resistance to EGFR-targeted agents: a systematic review and meta-analysis of studies in advanced non-small-cell lung cancer and metastatic colorectal cancer. The Lancet. Oncology.

[30] Laurentpuig P, Béroud C, Soussi T (1998). APC gene: database of germline and somatic mutations in human tumors and cell lines. Nucleic Acids Research.

[31] Carethers JM, Jung BH (2015). Genetics and Genetic Biomarkers in Sporadic Colorectal Cancer. Gastroenterology.

[32] Seo AN (2014). HER2 status in colorectal cancer: its clinical significance and the relationship between HER2 gene amplification and expression. PloS One.

[33] Marx AH (2010). Heterogenous high-level HER-2 amplification in a small subset of colorectal cancers. Human Pathology.

[34] Kavuri SM (2015). HER2 Activating Mutations Are Targets for Colorectal Cancer Treatment. Cancer Discovery.

[35] Slamon DJ (1989). Studies of the HER-2/neu proto-oncogene in human breast and ovarian cancer. Science.

[36] Gravalos C, Jimeno A (2008). HER2 in gastric cancer: a new prognostic factor and a novel therapeutic target. Annals of Oncology.

[37] Ross JS (2018). Targeting HER2 in colorectal cancer: The landscape of amplification and short variant mutations in ERBB2 and ERBB3. Cancer.

[38] Park DI (2007). HER-2/neu overexpression is an independent prognostic factor in colorectal cancer. International Journal of Colorectal Disease.

[39] Wang XY (2019). Significance of HER2 protein expression and HER2 gene amplification in colorectal adenocarcinomas. World Journal of Gastrointestinal Oncology.

