# Supplementary material for: Somatic mutation profiling and HER2 status in KRAS-positive Chinese colorectal cancer patients
Source: Sci Rep. 2019 Nov 15;9:16894. doi: 10.1038/s41598-019-53039-y (PMC6858340; doi:10.1038/s41598-019-53039-y)
Supplement: Supplementary file 1 — Somatic mutation profiling and HER2 status in KRAS-positive Chinese colorectal cancer patients [file 41598_2019_53039_MOESM1_ESM.pdf]

Somatic mutation profiling and *HER2* status in *KRAS*-positive Chinese colorectal cancer patients

Zhouhuan Dong[1,+], Linghong Kong[2,+], Zhiyi Wan[2], Fengwei Zhu[1], Mei Zhong[1], Yali Lv[1], Po Zhao[1], Huaiyin Shi[1,*]

[1] Chinese PLA General Hospital, Department of Pathology, Beijing, 100853, China

[2] ChuiYangLiu Hospital affiliated to Tsinghua University, Department of Pathology, Beijing, 100022, China

* Correspondence: shihuaiyin@sina.com

[+] These authors contributed equally to this work.

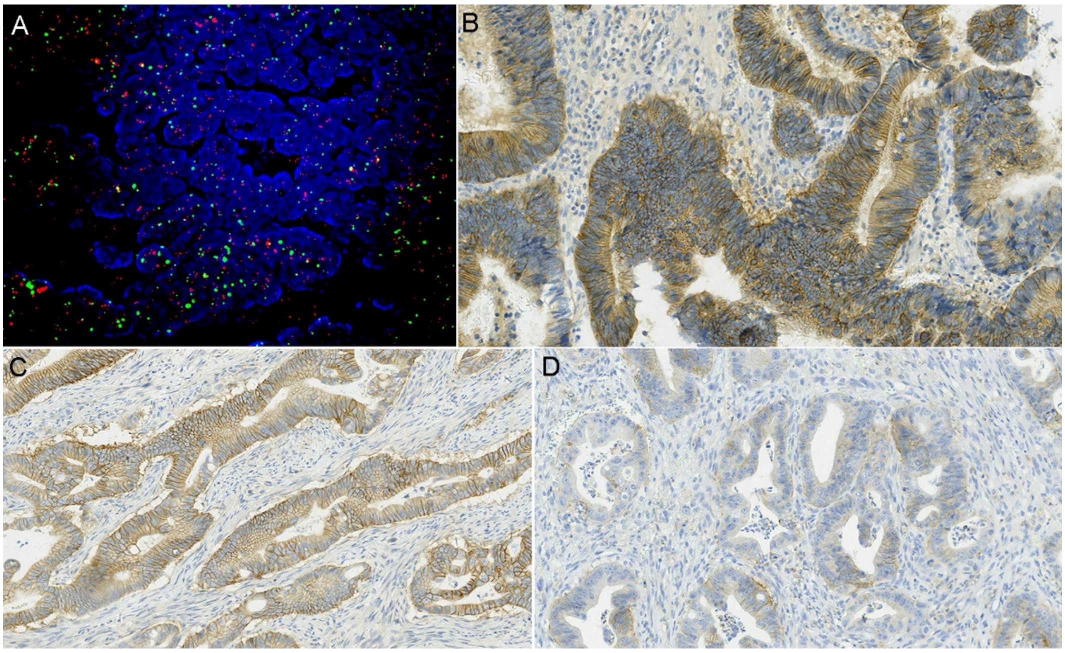

Figure S1. Representative figures of HER2 status by FISH and IHC analyses in CRC patients. A. *HER2* gene amplification by FISH (100 × objective), CEP17 is green-labeled; *HER2* gene is red-labeled. B. IHC 3+ (20 × objective). C. IHC 2+ (20 × objective). D. IHC 0/1+ (20 × objective).

Table S1. Gene list of the SGI OncoAim™ DNA Panel.

ABL1	AKT1	ALK	APC	ATM
BRAF	CBL	CDH1	CDK4	CDKN2A
CHEK2	CSF1R	CTNNB1	DNMT3A	EGFR
ERBB2	ERBB3	ERBB4	EZH2	FBXW7
FGFR1	FGFR2	FLT3	GNA11	GNAS
HNF1A	HRAS	IDH2	JAK1	JAK2
JAK3	KDR	KIT	KRAS	MET
MLH1	MPL	NFE2L2	NOTCH1	NPM1
NRAS	PAX5	PDGFRA	PIK3CA	PPP2R1A
PTCH1	PTEN	RAF1	RB1	RET
SF3B1	SMAD4	SMARCB1	SMO	STAT3
STK11	TP53	U2AF1	VHL	

Table S2. The distribution of all gene mutations.

Sample	NGS Data Analysis					
	Gene Symbol	Position	Frequency	Locus Name	cDNA Change	Codon Change
1	APC	chr5: 112175488	0.439	COSM4662393	c.4197delT	p.S1400fs*15
1	EGFR	chr7: 55249088	0.581	COSM20891	c.2386G>A	p.G796S
1	KRAS	chr12: 25398285	0.308	COSM517	c.34G>A	p.G12S
1	TP53	chr17: 7579321	0.525	COSM44882	c.365_366delTG	p.V122fs*26
2	KRAS	chr12: 25398285	0.47	COSM517	c.34G>A	p.G12S
2	TP53	chr17: 7578217	0.285	COSM43939	c.632C>T	p.T211I
3	APC	chr5: 112175676	0.301	COSM18873	c.4385_4386delAG	p.S1465fs*3
3	KRAS	chr12: 25398285	0.207	COSM517	c.34G>A	p.G12S
3	TP53	chr17: 7577568	0.324	COSM11059	c.713G>A	p.C238Y
4	APC	chr5: 112175423	0.161	COSM18862	c.4132C>T	p.Q1378*
4	IDH2	chr15: 90631924	0.181	COSM4385675	c.428_429insG	p.T146fs*126
4	KRAS	chr12: 25398285	0.152	COSM518	c.34G>C	p.G12R
4	PIK3CA	chr3: 178952085	0.204	COSM775	c.3140A>G	p.H1047R
5	KRAS	chr12: 25398285	0.287	COSM516	c.34G>T	p.G12C
5	TP53	chr17: 7576924	0.354	COSM99753	c.921_922delAC	p.L308fs*28
6	FBXW7	chr4: 153245447	0.157	COSM30602	c.1744T>C	p.S582P
6	KRAS	chr12: 25398285	0.254	COSM516	c.34G>T	p.G12C
6	PIK3CA	chr3: 178936091	0.208	COSM125370	c.1633G>A	p.E545K
6	TP53	chr17: 7577580	0.374	COSM10725	c.701A>G	p.Y234C
7	APC	chr5: 112175639	0.336	COSM13127	c.4348C>T	p.R1450*
7	KRAS	chr12: 25398284	0.456	COSM521	c.35G>A	p.G12D
7	SMAD4	chr18: 48591931	0.284	COSM1389067	c.1094G>A	p.G365D
7	TP53	chr17: 7578222	0.374	COSM13120	c.626_627delGA	p.R209fs*6
8	KRAS	chr12: 25398284	0.167	COSM521	c.35G>A	p.G12D
8	SMAD4	chr18: 48591918	0.139	COSM14140	c.1081C>T	p.R361C
8	TP53	chr17: 7578413	0.278	COSM121042	c.517G>C	p.V173L
9	APC	chr5: 112175982	0.4	COSM19478	c.4691T>G	p.L1564*
9	KRAS	chr12: 25398284	0.353	COSM521	c.35G>A	p.G12D
9	PIK3CA	chr3: 178921553	0.297	COSM754	c.1035T>A	p.N345K
9	TP53	chr17: 7578406	0.495	COSM10648	c.524G>A	p.R175H
10	APC	chr5: 112175952	0.152	COSM4381242	c.4661_4662insA	p.T1556fs*3
10	KRAS	chr12: 25398284	0.196	COSM521	c.35G>A	p.G12D
10	TP53	chr17: 7577107	0.284	COSM44972	c.831T>A	p.C277*
11	APC	chr5: 112175952	0.344	COSM4381242	c.4661_4662insA	p.T1556fs*3
11	KRAS	chr12: 25398284	0.295	COSM521	c.35G>A	p.G12D
11	PIK3CA	chr3: 178936094	0.308	COSM766	c.1636C>A	p.Q546K
11	TP53	chr17: 7578406	0.473	COSM10648	c.524G>A	p.R175H
12	ERBB3	chr12: 56482341	0.104	COSM160822	c.889G>T	p.D297Y
12	KRAS	chr12: 25398284	0.222	COSM521	c.35G>A	p.G12D
12	TP53	chr17: 7577586	0.232	COSM44601	c.695T>C	p.I232T
13	APC	chr5: 112175621	0.114	COSM19021	c.4330C>T	p.Q1444*
13	KRAS	chr12: 25398284	0.208	COSM521	c.35G>A	p.G12D
13	SMAD4	chr18: 48604754	0.194	COSM1151656	c.1576G>A	p.E526K
13	TP53	chr17: 7578394	0.223	COSM10889	c.536A>G	p.H179R
14	KRAS	chr12: 25398284	0.165	COSM521	c.35G>A	p.G12D
14	SMAD4	chr18: 48591918	0.102	COSM14140	c.1081C>T	p.R361C
15	KRAS	chr12: 25398284	0.211	COSM521	c.35G>A	p.G12D
16	KRAS	chr12: 25398284	0.158	COSM521	c.35G>A	p.G12D
16	NFE2L2	chr2: 178098803	0.113	COSM132957	c.242G>A	p.G81D

16	SMAD4	chr18: 48591918	0.127	COSM14140	c.1081C>T	p.R361C
16	TP53	chr17: 7576897	0.282	COSM10786	c.949C>T	p.Q317*
17	KRAS	chr12: 25398284	0.237	COSM521	c.35G>A	p.G12D
17	TP53	chr17: 7577548	0.29	COSM121035	c.733G>A	p.G245S
18	KRAS	chr12: 25398284	0.286	COSM521	c.35G>A	p.G12D
18	TP53	chr17: 7577121	0.074	COSM10659	c.817C>T	p.R273C
19	KRAS	chr12: 25398284	0.279	COSM521	c.35G>A	p.G12D
19	TP53	chr17: 7578413	0.082	COSM11084	c.517G>A	p.V173M
20	APC	chr5: 112175198	0.232	COSM13728	c.3907C>T	p.Q1303*
20	APC	chr5: 112175212	0.272	COSM18764	c.3921_3925delAAAAG	p.E1309fs*4
20	APC	chr5: 112175213	0.262	COSM19263	c.3922_3926delAAAGA	p.E1309fs*4
20	KRAS	chr12: 25398284	0.312	COSM521	c.35G>A	p.G12D
20	TP53	chr17: 7576927	0.299	COSM6917	c.920-1G>A	p.?
21	APC	chr5: 112175213	0.373	COSM13727	c.3922A>T	p.K1308*
21	KRAS	chr12: 25398284	0.227	COSM521	c.35G>A	p.G12D
22	KRAS	chr12: 25398284	0.267	COSM521	c.35G>A	p.G12D
22	TP53	chr17: 7579316	0.327	COSM1172515	c.370_371insG	p.C124fs*25
23	APC	chr5: 112175639	0.312	COSM13127	c.4348C>T	p.R1450*
23	KRAS	chr12: 25398284	0.275	COSM521	c.35G>A	p.G12D
23	SMAD4	chr18: 48591919	0.391	COSM14122	c.1082G>A	p.R361H
24	KRAS	chr12: 25398284	0.238	COSM521	c.35G>A	p.G12D
24	TP53	chr17: 7577548	0.122	COSM121035	c.733G>A	p.G245S
25	APC	chr5: 112175639	0.199	COSM13127	c.4348C>T	p.R1450*
25	KRAS	chr12: 25398284	0.165	COSM522	c.35G>C	p.G12A
26	APC	chr5: 112175952	0.255	COSM4381242	c.4661_4662insA	p.T1556fs*3
26	KRAS	chr12: 25398284	0.23	COSM522	c.35G>C	p.G12A
26	PIK3CA	chr3: 178952085	0.221	COSM775	c.3140A>G	p.H1047R
27	APC	chr5: 112175198	0.176	COSM13728	c.3907C>T	p.Q1303*
27	KRAS	chr12: 25398284	0.167	COSM520	c.35G>T	p.G12V
27	TP53	chr17: 7578235	0.179	COSM44169	c.614A>C	p.Y205S
28	KRAS	chr12: 25398284	0.452	COSM520	c.35G>T	p.G12V
28	TP53	chr17: 7577115	0.525	COSM45251	c.823delT	p.C275fs*70
29	KRAS	chr12: 25398284	0.225	COSM520	c.35G>T	p.G12V
29	PIK3CA	chr3: 178952085	0.209	COSM775	c.3140A>G	p.H1047R
30	KRAS	chr12: 25398284	0.288	COSM520	c.35G>T	p.G12V
30	TP53	chr17: 7578406	0.461	COSM10648	c.524G>A	p.R175H
31	KRAS	chr12: 25398284	0.291	COSM520	c.35G>T	p.G12V
31	TP53	chr17: 7577548	0.32	COSM121035	c.733G>A	p.G245S
32	KRAS	chr12: 25398284	0.252	COSM520	c.35G>T	p.G12V
33	KRAS	chr12: 25398284	0.231	COSM520	c.35G>T	p.G12V
33	PIK3CA	chr3: 178916944	0.26	COSM13570	c.331A>G	p.K111E
33	TP53	chr17: 7578212	0.342	COSM10654	c.637C>T	p.R213*
34	KRAS	chr12: 25398284	0.216	COSM520	c.35G>T	p.G12V
34	PIK3CA	chr3: 178936091	0.201	COSM125370	c.1633G>A	p.E545K
34	SMAD4	chr18: 48591919	0.254	COSM14122	c.1082G>A	p.R361H
35	APC	chr5: 112175213	0.527	COSM13727	c.3922A>T	p.K1308*
35	KRAS	chr12: 25398284	0.456	COSM520	c.35G>T	p.G12V
36	APC	chr5: 112175639	0.251	COSM13127	c.4348C>T	p.R1450*
36	KRAS	chr12: 25398284	0.173	COSM520	c.35G>T	p.G12V
36	TP53	chr17: 7578211	0.232	COSM241997	c.638G>T	p.R213L
37	ERBB3	chr12: 56478854	0.202	COSM20710	c.310G>A	p.V104M

37	KRAS	chr12: 25398281	0.176	COSM532	c.38G>A	p.G13D
38	KRAS	chr12: 25398281	0.624	COSM532	c.38G>A	p.G13D
38	TP53	chr17: 7577548	0.298	COSM121035	c.733G>A	p.G245S
38	TP53	chr17: 7578479	0.304	COSM10905	c.451C>T	p.P151S
39	APC	chr5: 112175651	0.282	COSM2991034	c.4360delA	p.N1455fs*18
39	KRAS	chr12: 25398281	0.4	COSM532	c.38G>A	p.G13D
40	APC	chr5: 112175676	0.148	COSM1432411	c.4384_4385insAG	p.S1465fs*9
40	APC	chr5: 112175952	0.188	COSM19000	c.4661delA	p.T1556fs*9
40	KRAS	chr12: 25398281	0.171	COSM532	c.38G>A	p.G13D
41	KRAS	chr12: 25398281	0.305	COSM532	c.38G>A	p.G13D
41	TP53	chr17: 7577538	0.596	COSM10662	c.743G>A	p.R248Q
42	APC	chr5: 112175952	0.16	COSM4381242	c.4661_4662insA	p.T1556fs*3
42	ATM	chr11: 108142000	0.209	COSM1297603	c.2944C>T	p.R982C
42	CTNNB1	chr3: 41266136	0.478	COSM5663	c.133T>C	p.S45P
42	KRAS	chr12: 25398281	0.22	COSM532	c.38G>A	p.G13D
43	FBXW7	chr4: 153258983	0.233	COSM22971	c.832C>T	p.R278*
43	KRAS	chr12: 25398281	0.117	COSM532	c.38G>A	p.G13D
44	APC	chr5: 112175639	0.184	COSM13127	c.4348C>T	p.R1450*
44	JAK1	chr1: 65312344	0.164	COSM3734693	c.1975C>T	p.R659C
44	KRAS	chr12: 25398284	0.148	COSM521	c.35G>A	p.G12D
44	PIK3CA	chr3: 178927980	0.126	COSM757	c.1258T>C	p.C420R
44	TP53	chr17: 7574003	0.169	COSM11073	c.1024C>T	p.R342*
44	TP53	chr17: 7574018	0.183	COSM11071	c.1009C>T	p.R337C
45	KRAS	chr12: 25398284	0.16	COSM521	c.35G>A	p.G12D
45	PIK3CA	chr3: 178952085	0.09	COSM775	c.3140A>G	p.H1047R
46	APC	chr5: 112175490	0.401	COSM19044	c.4199C>A	p.S1400*
46	KRAS	chr12: 25398281	0.4	COSM532	c.38G>A	p.G13D
46	TP53	chr17: 7578265	0.405	COSM11089	c.584T>C	p.I195T
47	APC	chr5: 112175240	0.245	COSM19253	c.3949G>T	p.E1317*
47	KRAS	chr12: 25398284	0.253	COSM521	c.35G>A	p.G12D
47	PIK3CA	chr3: 178952007	0.275	COSM12461	c.3062A>G	p.Y1021C
48	APC	chr5: 112175952	0.205	COSM4381242	c.4661_4662insA	p.T1556fs*3
48	KRAS	chr12: 25398284	0.2	COSM521	c.35G>A	p.G12D
48	TP53	chr17: 7578263	0.195	COSM10705	c.586C>T	p.R196*
49	ERBB3	chr12: 56478854	0.237	COSM160824	c.310G>C	p.V104L
49	KRAS	chr12: 25398284	0.22	COSM521	c.35G>A	p.G12D
50	KRAS	chr12: 25398281	0.383	COSM532	c.38G>A	p.G13D
50	TP53	chr17: 7577121	0.441	COSM10659	c.817C>T	p.R273C

Table S3. HER2 status assessed by FISH and IHC in 139 primary CRC patients.

	IHC score			
	Negative		Equivocal	Positive
	0	1 +	2 +	3+
FISH +	0	1	6	4
FISH −	13	48	63	4
Total (%)	13（9.35）	49（35.25）	69（49.64）	8（5.76）
